# Low level of isocitrate dehydrogenase 1 predicts unfavorable postoperative outcomes in patients with clear cell renal cell carcinoma

**DOI:** 10.1186/s12885-018-4747-1

**Published:** 2018-08-28

**Authors:** Pingcuo Laba, Jianfeng Wang, Jin Zhang

**Affiliations:** 1 Department of Urology, Shigatse People’s Hospital, Shigatse, 85700 China; 2 Department of Urology, Renji Hospital, School of Medicine, Shanghai Jiaotong University, No.160, Pujian Road, Shanghai, 200127 China

**Keywords:** Clear-cell renal cell carcinoma, Isocitrate dehydrogenase 1, Prognosis, Overall survival, Recurrence-free survival

## Abstract

**Background:**

The purpose of this study was to investigate the role of isocitrate dehydrogenase 1 (IDH1) expression on prognosis of patients with clear cell renal cell carcinoma (ccRCC) following nephrectomy.

**Methods:**

We retrospectively enrolled 358 ccRCC patients undergoing nephrectomy in Renji Hospital. Clinicopathologic features, overall survival (OS) and recurrence-free survival (RFS) of ccRCC patents were all collected. IDH1 expression level was assessed by immunohistochemistry and its association with clinicopathologic features and outcomes were also evaluated. Kaplan-Meier method with the log-rank test was applied to compare survival curves. Multivariate cox regression models were applied to analyze the prognostic value of each factor on OS and RFS of ccRCC patients. Moreover, two nomograms with factors selected by multivariate analysis were constructed to evaluate the prognosis of ccRCC patients, and the calibration plots were built to assess the predictive accuracy of nomograms.

**Results:**

Our data indicated that IDH1 expression level was down-regulated in ccRCC tissues, and it negatively correlated with tumor Fuhrman grade (*p* = 0.025). Low IDH1 expression was associated with worse OS and RFS for cccRCC patients (OS, *p* = 0.004; RFS, *p* = 0.03). In addition, IDH1 could significantly stratify patients’ OS and RFS in intermediate/high risk patients (UISS score ≥ 4) (*p* = 0.049 and *p* = 0.004, respectively). Furthermore, incorporating IDH1 with other prognostic factors could predict ccRCC patients’ OS and RFS (OS, c-index = 0.779; RFS, c-index = 0.798) and perform better than TNM and SSIGN system.

**Conclusions:**

Low IDH1 expression level might be an adverse prognostic biomarker for clinical outcomes of ccRCC patients, and two nomograms with IDH1 are potential effective prognostic models for ccRCC.

## Background

Renal cell carcinoma (RCC) is the most common type of malignant tumor in kidney, which represents the sixth most common cancer in men and the tenth most common cancer in women [1]. Clear cell RCC (ccRCC) is the prominent histological subtype of RCC, which accounts for about 80% to 90% of all RCC patients [2]. However, despite nephrectomy may cure the majority of localized RCC, about one third of the patients undergo local recurrence or distant metastasis after nephrectomy [3]. Currently, several clinical or pathological outcome prediction systems have already been established to evaluate the outcomes of patients with RCC, such as the University of California Los Angeles integrated staging system (UISS), the Mayo clinic stage, size, grade, and necrosis score (SSIGN), and the TNM stage, Fuhrman grade, Eastern Cooperative Oncology Group performance status (ECOG PS) [4–6]. However, due to the heterogeneity of molecular phenotype, there is a long way to go to predict the clinical outcomes of ccRCC [7, 8]. Therefore, new prognosis prediction systems with high accuracy are imperative needed for patients with ccRCC.

Altered cellular metabolism in cancers was observed for many years [9]. Despite the presence of enough oxygen, cancer cells still show high levels of glycolysis, which means the altered cell metabolic regulation plays an important role in tumorigenesis [10]. Isocitrate dehydrogenases (IDHs) are comprised of three members: IDH1, IDH2 and IDH3 [11]. IDH1 mainly catalyzes the conversion of isocitrate to alpha-ketoglutarate (aKG), and provides sufficient NADPH and regulates the biosynthesis of cholesterol and fatty acid [12]. Recently, many studies showed that IDH1 is mutated in various human cancers, especially in low-grade glioma [13]. The most common mutation site of IDH1 is the R132H, which acquires the ability to catalyze the reduction of a-KG to 2-hydroxyglutarate (2-HG) [14]. Moreover, several studies indicated that the R132H mutation of IDH1 correlates with a favorable prognosis for patients with glioma and gastrointestinal cancer [15, 16]. Nevertheless, little research was conducted to investigate the relationship between wide-type IDH1 and tumors. Sun and colleagues reported that IDH1 was significantly higher in patients with non-small cell lung cancer than in healthy controls [17]. However, the exact role of IDH1 in ccRCC, especially patients with high risk, is remains unknown.

The aim of this study was to reveal the clinical role of IDH1 in ccRCC patients. Moreover, two prognostic nomograms integrating IDH1 expression and clinical factors were established to predict the outcomes and may guide clinical decisions making for ccRCC patients.

## Methods

### Patients

A total of 358 ccRCC patients who underwent nephrectomy in the Department of Urology, Renji Hospital, during Jan 2005 and Dec 2008, were retrospectively included in our study. Patients’ clinicopathologic information, including gender, age, TNM stage, Fuhrman grade, tumor size, presence of tumor necrosis and SSIGN were collected. TNM stage was determined by one senior urologist according to the 2010 AJCC (the American Joint Committee on Cancer) TNM classification [18]. Fuhrman grade and tumor necrosis were determined according to the 2014 EAU guidelines and 2012 ISUP (International Society of Urological Pathology) consensus [19]. The SSIGN score were applied to stratify patient risks, just as previously reported [6]. OS and RFS were calculated as previously reported [20]. The last follow-up was at Apr. 30, 2016.

### Tissue microarray (TMA) and immunohistochemistry

Tissue microarray (TMA) was constructed based on all the patients’ tumor samples, and were stained according to the standard method [21]. Primary antibody against human IDH1 (diluted:1:300; Abcam) was applied for immunohistochemistry (IHC) staining. Visualization reagent (Nikon eclipse Ti Microscope) was used to record the results. Tissue staining intensity and percentage were scored. Five areas of IDH1 positive stains were selected to estimate at low (× 40) or high (× 200) magnification. The intensity was scored as: 0 (negative), 1 (weak), 2 (moderate), and 3 (strong); the percentage of cells was scored into the following four categories: 1 (0–25%), 2 (26–50%), 3 (51–75%), or 4 (> 75%), and comprehensive score = staining percentage × intensity [22]. Finally, the expression level of IDH1 was defined into low expression and high expression according to the comprehensive score (cutoff value = 6) [23].

### Statistical analysis

SPSS Statistics 22.0 and R software were used to performed statistics. Akaike information criterion (AIC) and Harrell’s concordance index (C-index) value were used to assess the predictive accuracy and sufficiency of different models [24]. Nomograms and calibration plots were generated by R software with “rms” package, and parameters included in nomograms were based on statistical significance in multivariate analysis. Two-sided *p* value < 0.05 was considered as statistically significant.

## Results

### Patients’ characteristics and association with IDH1 expression

**Fig. 1:**
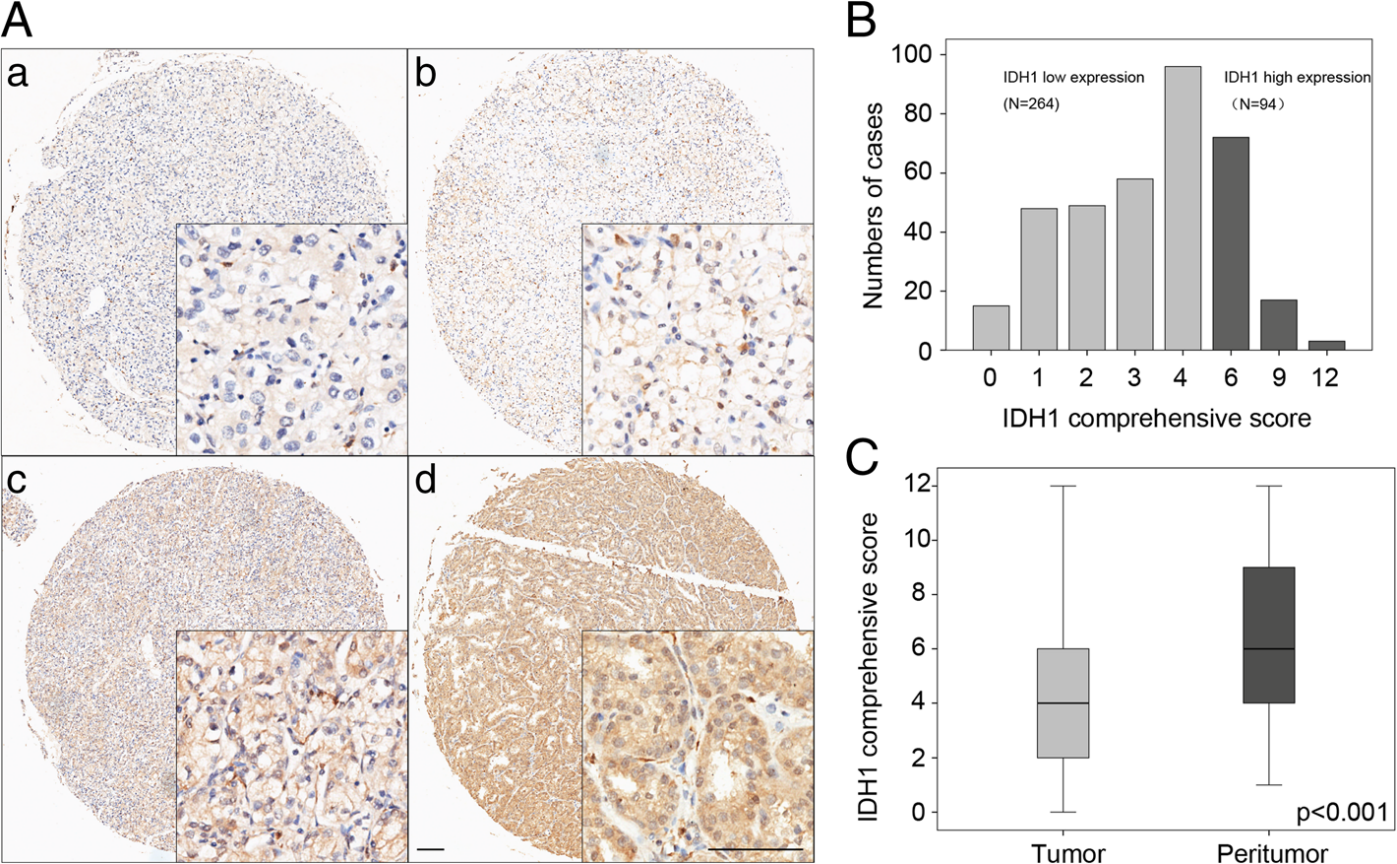
**A** Representative immunohistochemical staining of IDH1 in ccRCC tissues. **A** (a). Negative staining of IDH1 in ccRCC, score 0. **A** (b). Weak staining of IDH1 in ccRCC, score 3. **A** (c). Moderate staining of IDH1 in ccRCC, score 6. **A** (d). Strong staining of IDH1 in ccRCC, score 12. Bar: 100 μm. **B** Frequency distribution of tumoral IDH1 immunohistochemistry integrated comprehensive score in 358 ccRCC samples. **C** Comprehensive score of IDH1 expression level in tumor and peritumor tissues. *p* value < 0.05 was regarded as statistically significant

Patients’ clinical characteristics are shown in Table 1. To evaluate the expression level of IDH1 in ccRCC tumor tissues, IHC staining analysis was used in TMAs among all the ccRCC patients. IDH1 expression was predominantly found in the cytoplasm of tumor cells, with variable staining intensity in different specimens (Fig. 1A and B). Moreover, IDH1 was found to be down-regulated in ccRCC tumor tissues, compared with peritumor tissues (Fig. 1C, *p* < 0.001). After IHC evaluation, study cohort was divided into two groups: low IDH1 expression group (264 patients) and high IDH1 group (94 patients). The association between patients’ clinicopathological features and the IDH1 expression level are summarized in Table 1. IDH1 expression level in tumor tissues was negatively correlated with tumor Fuhrman grade (*p* = 0.025), whereas other patients’ clinicopathological features, including gender, age, TNM stage, T stage, N stage, M stage, tumor size, necrosis and SSIGN score, were not correlated with IDH1 expression (*p* > 0.05).

**Table 1:** Association of IDH1 expression with clinicopathological characteristics in ccRCC patients

Characteristics	Patients		IDH1 expression		
	n	%	Low	High	*p*-value
All patients	358	100	264	94	
Gender					
Male	254	70.9	189	65	0.654^a^
Female	104	29.1	75	29	
Age(years)					
≤ 55	178	49.7	125	53	0.132^a^
> 55	180	50.3	139	41	
TNM stage					
I	286	79.9	208	78	0.384^a^
II + III + IV	72	20.1	56	16	
T stage					
T1 + T2	344	96.1	253	91	1.000^b^
T3 + T4	14	3.9	11	3	
N stage					
N0	349	97.5	256	93	0.455^b^
N1	9	2.5	8	1	
M stage					
M0	352	983	260	92	0.655^b^
M1	6	1.7	4	2	
Fuhrman grade					
I + II	297	83.0	212	85	0.025^a*^
III + IV	61	17.0	52	9	
Tumor size(cm)					
≤ 5	186	52.0	134	52	0.447^a^
> 5	172	48.0	130	42	
Necrosis					
Absent	296	82.7	216	80	0.469^a^
Present	62	17.3	48	14	
SSIGN score					
< 4	266	74.3	193	73	0.386^a^
≥ 4	92	25.7	71	21	

^a^Chi-square test

^b^Fisher’s exact test

*p < 0.05 indicates a significant association among the variables

### Low IDH1 expression is associated with poor prognosis

**Fig. 2:**
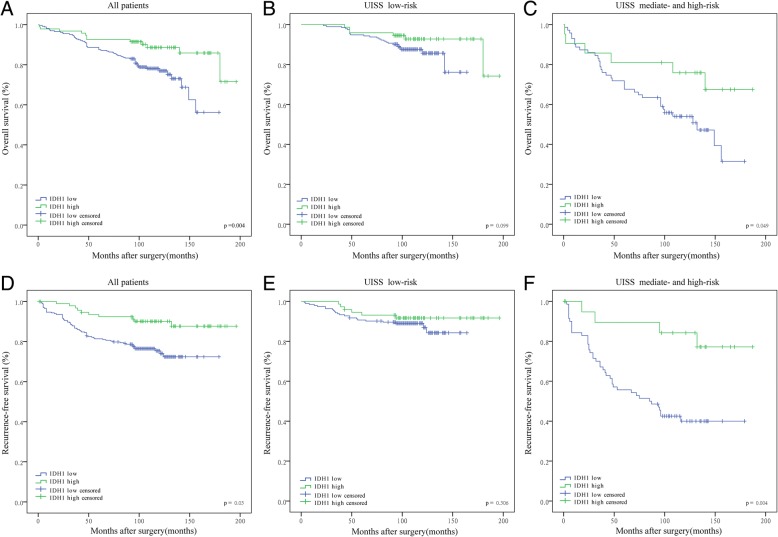
Tumoral IDH1 expression stratified by SSIGN score and related Kaplan-Meier analyses of patient overall survival (OS) and recurrence free survival (RFS). **a** OS of all ccRCC patients according to IDH1 expression; (**b-c**) OS of patients in different SSIGN risk groups according to IDH1 expression; (**d**) RFS of all ccRCC patients according to IDH1 expression; (**e-f**) RFS of patients in different SSIGN risk groups according to IDH1 expression. *p* value < 0.05 was regarded as statistically significant

Survival curves were performed to compare OS and RFS of ccRCC patients according to IDH1 expression. Interesting, patients with low IDH1 expression showed poorer OS (*p* = 0.004) and RFS (*p* = 0.03) than patients in high IDH1 expression group (Fig. 2a and d). Moreover, patients was classify into two groups: 0–3 (low-risk group), ≥4 (intermediate- and high-risk group) by SSIGN score. As is shown in Fig. 2c and f, IDH1 expression level showed as an adverse prognostic factor for both OS and RFS in intermediated- and high-risk groups (OS, *p* = 0.049; RFS, *p* = 0.004), while in the low-risk group it did not meet statistical significance.

Further, multivariate analysis was used to assess whether IDH1 expression level is an independent prognostic factor for outcomes of ccRCC patients. As presented in Table 2, low IDH1 expression in tumor was an unfavorable independent predictor for OS and RFS of ccRCC patients (OS, HR, 0.500, 95% CI, 0.253–0.987, *p* = 0.046; RFS, HR, 0.463, 95% CI, 0.233–0.922, *p* = 0.028). In addition, TNM stage, N stage, Fuhrman grade, and tumor size were also considered as independent predictors for both OS and RFS.

**Table 2:** Multivariate cox regression analysis for overall survival and recurrence-free survival in ccRCC patientsᅟ

Variables	Overall survival			Recurrence-free survival		
	HR	(95% CI)	p*	HR	(95% CI)	p*
IDH1 in cancer tissues						
Low	1			1		
High	0.500	0.253–0.987	0.046*	0.463	0.233–0.922	0.028*
TNM stage						
I	1			1		
II + III + IV	3.866	1.185–12.610	0.025*	6.453	1.645–25.309	0.007*
T stage						
T1 + T2	1			1		
T3 + T4	2.991	1.197–7.473	0.019*	2.076	0.901–4.785	0.086
N stage						
N0	1			1		
N1	3.884	1.418–10.640	0.008*	3.319	1.253–8.792	0.016*
Fuhrman grade						
I + II	1			1		
III + IV	2.255	1.314–3.869	0.003*	2.069	1.203–3.557	0.009*
Tumor size(cm)						
≤ 4	1			1		
> 4	3.261	1.584–6.712	0.001*	3.482	1.658–7.313	0.001*

*HR* hazard ratio, *95% CI* 95% confidence interval, *p < 0.05 was considered statistically significant

### Comparison of the predictive abilities between IDH1 expression and other prognostic factors

To investigate the predictive ability of IDH1 expression in ccRCC, IDH1 was compared with several conventional ccRCC prognosis predictors, such as SSIGN outcome algorithm, TNM stage, T stage, N stage, Fuhrman grade and tumor sizes. As showed in Table 3, the C-indexes of IDH1 were 0.566 and 0.579 for OS and RFS respectively, which were higher than N stage (0.563and 0.55) and lower than SSIGN outcome algorithm, TNM stage, N stage, Fuhrman grade and tumor sizes. Moreover, the C-index of those models was increased when IDH1 expression factor was replenished both for OS and RFS, suggesting the expression level of IDH1 has a good predictive ability for ccRCC outcomes. Besides, the AIC value of all factors integrated model was lower than SSIGN outcome algorithm, which means the model integrated with all factors performed better than SSIGN to predict ccRCC prognosis.

**Table 3:** Comparison of the predictive accuracies of prognostic factorsᅟ

Model	Overall Survival (*N* = 285)		Recurrence free survival (*N* = 285)	
	C-Index	AIC	C-Index	AIC
IDH1	0.566	798.932	0.579	833.571
TNM stage	0.694	746.2725	0.724	779.0914
TNM stage+IDH1	0.722	738.6321	0.749	769.0795
T stage	0.686	750.3168		
T stage+IDH1	0.716	738.1326		
N stage	0.563	777.9406	0.55	819.1828
N stage+IDH1	0.62	772.8811	0.615	813.6082
Fuhrman grade	0.663	781.1461	0.656	818.4013
Fuhrman grade + IDH1	0.678	777.1598	0.68	813.3012
Tumor size	0.683	766.2329	0.703	793.0312
Tumor size+IDH1	0.712	759.9979	0.738	785.7259
Nomogram	0.779	716.8505	0.798	749.2201
SSIGN	0.748	732.8423	0.77	764.8564

### Prognostic nomogram of ccRCC patients

**Fig. 3:**
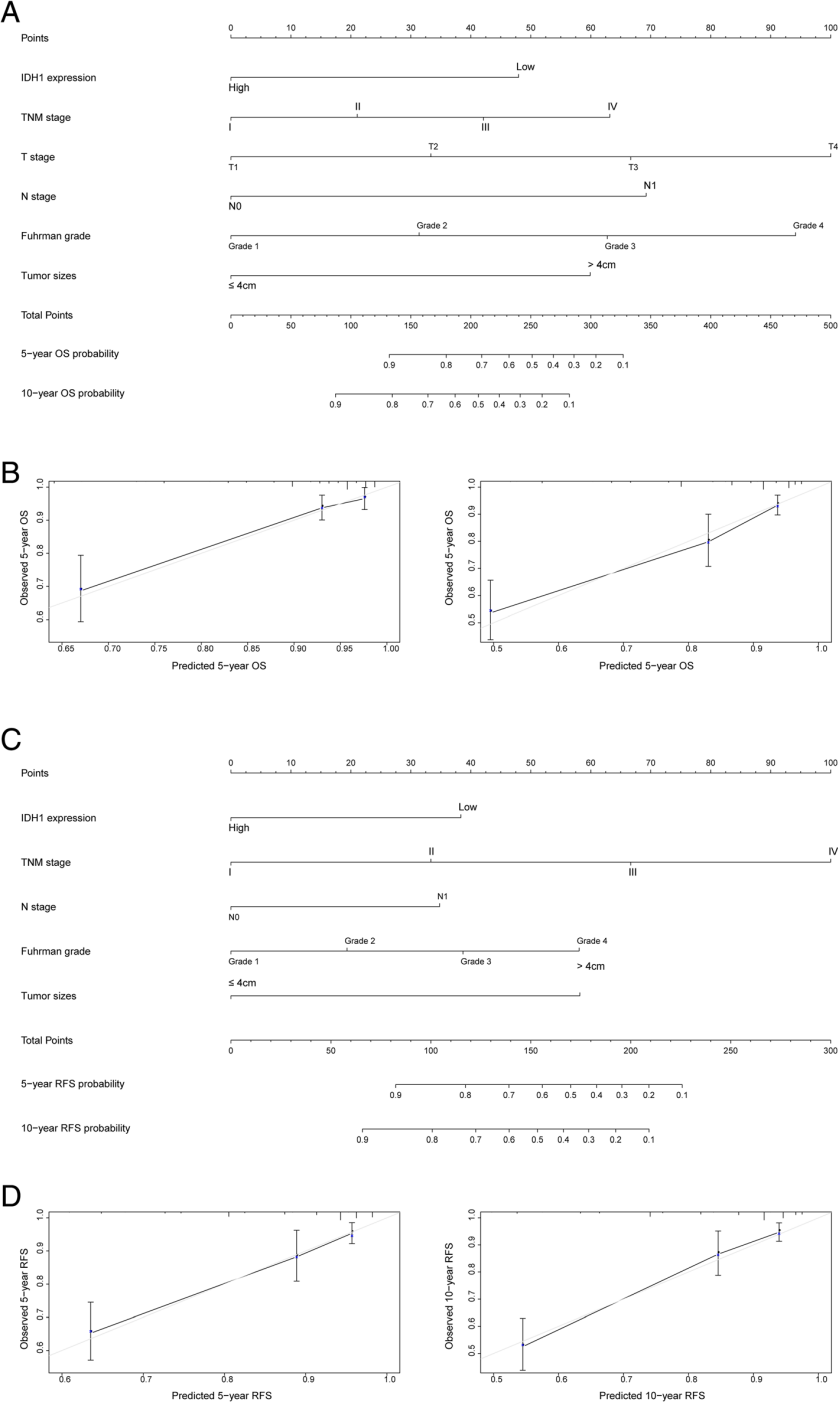
Nomograms and calibration plots for prognosis of OS and RFS in patients with ccRCC. **a** Nomogram for predicting the OS of ccRCC patients; (**b**) The calibration plots for overall survival at 5 and 10 years. **c** Nomogram for predicting the RFS of ccRCC patients; (**d**) The calibration plots for overall survival at 5 and 10 years

In order to use IDH1 as a prognostic factor, two nomograms were constructed to predict prognosis of ccRCC patients, via integrating the independent risk factors from multivariate analysis [25]. In addition, calibration plots of the nomograms for OS and RFS at 5 and 10 year revealed the good consistency between the actual and predicted survival of patients **(**Fig. 3**)**. Our results suggest that these nomograms may be reliable prognostic models for ccRCC patients.

## Discussion

IDH1, a NADP-dependent enzyme which involves in the control of oxidative cellular damage, was identified as a tumor suppressor since its inactivation plays a vital role in tumorigenesis [26, 27]. Although IDH1 have consistently been reported take part in the genesis of many cancers, the correlation between IDH1 expression and ccRCC outcomes remains unclear. In this study, we detected IDH1 was mainly expressed in the cytoplasm of ccRCC tumor cells, and low expression level of IDH1 in tumor correlated with an adverse outcomes of ccRCC, especially in patients with high SSIGN scores. Besides, IDH1 expression level was a risk factor of OS and RFS for ccRCC patients. Furthermore, by integrating with several factors from multivariate analysis, IDH1 expression in tumors could enhance the prognostic accuracy of these factors, including SSIGN outcome algorithm, TNM stage, N stage, Fuhrman grade and tumor sizes. Moreover, two prognostic nomograms models were constructed to predict OS and RFS of ccRCC patients, via integrating IDH1 with significant factors based on the multivariate analysis. Further c-index analysis indicated two prognostic nomograms had better prognostic capability compared with SSIGN prognostic model.

In mammal cells, three types of IDHs were discovered, including IDH1, IDH2, and IDH3. Although these three enzymes show the similar enzymatic reaction, they have different functions in different places. IDH1 performs its enzymatic activity mainly in the cytosol and the peroxisomes [28]. In recent years, mutations in IDH1 gene were reported in various tumors, which confer IDH1 the new enzymatic function of catalyzing α-KG to R-2-hydroxyglutarate (2-HG) [29]. The role of IDH1 gene mutation in esophageal squamous cell carcinoma [30], glioma [31] and acute myeloid leukemia (AML) have been successively reported [32]. The expression level of IDH1-R132H, which is the most common mutation type, correlates with poor outcomes in several cancers, such as gastrointestinal cancer and nonenhancing diffuse glioma [16, 33]. Besides, IDH1 wide-type expression was also explored in tumors. Overexpression of IDH1 was reported in non-small cell lung (NSCL) cancer, both in plasma and tumor tissues [17, 34]. However, our study revealed that low expression of IDH1 was associated with adverse ccRCC patients’ prognosis, which was inconsistent with that in NSCL cancer. Similarly, down-regulation of IDH1 was detected in kidney cancer [35], which means the loss of IDH1 expression might contribute ccRCC genesis.

The mechanism of low IDH1 participates in progression of cancers has not been well elaborated. IDH1 not only plays an important role on the biosynthesis of central metabolites in the tricarboxylic acid (TCA) cycle, but represents the major pathway for cellular NADPH generation in cells, a vital factor that regulates the amount of glutathione (GSH) and thioredoxin in cells [36]. GSH and thioredoxin are the main members of antioxidative systems, which could protect cells from oxidative damages by eliminating the reactive oxygen species (ROS) [37, 38]. Thus, loss the enzymatic function of IDH1 in tumor cells could impair detoxification procedure, which may result in DNA damages and genes mutations [39]. Besides, IDH could also regulate cellular apoptosis, and facilitate the development of a modifier of cancer chemotherapy [40].

Although the clinical significance of IDH1 in ccRCC was revealed, several limitations still exist in our study. Firstly, it is a retrospective cohort in a single center with limited patients, especially for the patients with advanced ccRCC. Therefore, patients from multicentric cohort are necessary to confirm our findings. Secondly, our study was based on the IHC staining and scored by two pathologists, and it would be more persuasive by measuring the mRNA or protein expression level of IDH1. Another problem is that, the time of patients follow up is not long enough to illustrate the clinical significance of IDH1, and longer follow up time is in need. Thus, more researches, especially mechanism studies, are needed to further understand the role of IDH1 in ccRCC progression.

## Conclusions

In conclusion, we revealed low expression of IDH1 was significantly associated with poor ccRCC prognosis and could be used as a prognostic parameter to predict ccRCC patients’ outcomes. We also constructed two nomograms for ccRCC patients, which might be adapted into risk scoring systems and guide in clinical decisions for ccRCC patients.

## Abbreviations

AIC: Akaike information criterion
AJCC: American joint committee on cancer
ccRCC: Clear cell renal cell carcinoma
CI: Confidence interval
C-index: Concordance index
ECOG PS: Eastern Cooperative Oncology Group performance status
HR: Hazard ratio
IDH1: Isocitrate dehydrogenase 1
ISUP: International society of urological pathology
NADPH: Nicotinamide adenine dinucleotide phosphate
OS: Overall survival
RFS: Recurrence-free survival
SSIGN: Mayo clinic stage, size, grade and necrosis
TCA: tricarboxylic acid
TMA: Tissue microarray
UISS: University of California Integrated Staging System
